# The Evolution of Mentorship Capacity Development in Low- and Middle-Income Countries: Case Studies from Peru, Kenya, India, and Mozambique

**DOI:** 10.4269/ajtmh.18-0560

**Published:** 2018-11-14

**Authors:** Emilia Noormahomed, Pamela Williams, Andrés G. Lescano, Tony Raj, Elizabeth A. Bukusi, Robert T. Schooley, Craig R. Cohen

**Affiliations:** 1 Department of Microbiology, Faculty of Medicine, Universidade Eduardo Mondlane, Maputo, Mozambique;; 2 Division of Infectious Diseases, Department of Medicine, University of California, San Diego, California;; 3 Mozambique Institute for Health Education and Research, Maputo, Mozambique;; 4 Institute for Global Health Sciences, University of California, San Francisco, San Francisco, California;; 5 Emerge, Emerging Diseases and Climate Change Research Unit, Facultad de Salud Pública y Administración, Universidad Peruana Cayetano Heredia, Lima, Peru;; 6 St. John’s Research Institute, Bangalore, India;; 7 Research Care Training Program, Center for Microbiology Research, Kenya Medical Research Institute, Nairobi, Kenya;; 8 Departments of Global Health and Obstetrics and Gynecology, University of Washington, Seattle, Washington;; 9 University of California Global Health Institute, San Francisco, California

## Abstract

Following the Fogarty International Center-supported “Mentoring the Mentors” workshops in South America, Africa, and Asia, approaches and guidelines for mentorship at institutions within these low- and middle-income country (LMIC) contexts, appropriate for the respective regional resources and culture, were implemented. Through the presentation of case studies from these three geographic regions, this article illustrates the institutional mentorship infrastructure before the workshop and the identified gaps used to implement strategies to build mentorship capacity at the Universidad Peruana Cayetano Heredia (Peru), Kenya Medical Research Institute (Kenya), Saint John’s Research Institute (India), and Eduardo Mondlane University (Mozambique). These case studies illustrate three findings: first, that mentorship programs in LMICs have made uneven progress, and institutions with existing programs have exhibited greater advancement to their mentoring capacity than institutions without formal programs before the workshops. Second, mentoring needs assessments help garner the support of institutional leadership and create local ownership. Third, developing a culture of mentorship that includes group mentoring activities at LMIC institutions can help overcome the shortage of trained mentors. Regardless of the stage of mentoring programs, LMIC institutions can work toward developing sustainable, culturally effective mentorship models that further the partnership of early career scientists and global health.

## Introduction

Effective mentorship is key to the development, success, and retention of early career investigators in research settings.^1^ Successful mentorship requires not just skilled mentors, but formal mentorship training to both navigate the mentor–mentee relationship and provide guidance on methods to attain institutional support.^2,3^

This article describes the development, implementation, and evaluation of mentorship initiatives at four institutions before and after the “Mentoring the Mentors in Global Health Research” workshops. Through the presentation of case studies from the geographic regions of South America, Africa, and Asia, this article illustrates the institutional mentorship infrastructure before the workshop and the identified gaps used to implement strategies to build mentoring capacity at the Universidad Peruana Cayetano Heredia (UPCH) (Peru), Kenya Medical Research Institute (KEMRI) (Kenya), Saint John’s Research Institute (SJRI) (India), and Eduardo Mondlane University (Mozambique). The workshops were sponsored by the six consortia funded by the Fogarty International Center Global Health Program for Fellows and Scholars and were developed collaboratively between U.S.-based investigators with expertise in mentoring and senior faculty and academic leaders at academic institutions in South America, East Africa, South Asia and Southern Africa.^4–6^ The goal of these workshops was to train mid-and senior-level investigators conducting public health, social, clinical, and basic science research across multiple academic institutions in low- and middle-income countries (LMICs) to be more effective mentors and inculcate a culture of mentorship at their institutions. These case studies describe the mentoring environment of the institutions before the workshops and the degree the workshops and other factors influenced the establishment of formal mentorship at the respective institutions. In the absence of both established models and documentation of culturally effective mentorship, these case studies provide insights to an area of little prior recorded research.

## Study Design and Methods

A multiple, explanatory/qualitative case-study design was followed. The analysis unit was defined at the country level, but bounded by the relationship with the “Mentoring the Mentor” regional workshops and other key mentoring initiatives.^1^ Key informants (Emilia Noormahomed, Andrés G. Lescano, Tony Raj, and Elizabeth A. Bukusi) with active roles in the organization of the workshops and in future mentorship efforts were asked to generate a timeline of all major developments and outcomes produced as a direct or indirect consequence of the workshop, and identify gaps, lessons learned, and best practices. Initial timelines were developed during a consultative workshop held April 2017 that included most of the co-authors. The primary analysis approach attempted to capture the heterogeneity in the settings and strategies instead of comparing experiences head-to-head. Accounts provided by the key informants were complemented with web searches in an explanation-building approach. Cases were presented separately and an attempt was made to capture commonalities when available.^7^

## Case 1—UPCH, Peru

Universidad Peruana Cayetano Heredia is a private, not-for-profit university, and the leading research institution in the biomedical sciences in Peru,^8^ and plays a prominent role in South America. It was founded in 1961 and currently has more than 2,000 students spread across eight schools, including medicine, sciences, and public health. Universidad Peruana Cayetano Heredia is an important regional hub for international collaboration and leads numerous research and training grants from the U.S. National Institutes of Health, the Wellcome Trust, and other global sponsors, in addition to extensive international partnerships with centers across the globe. Its faculty and investigators include world leaders in their fields and professionals appointed to prominent public positions in government.

### Mentorship infrastructure before the 2013 workshop.

Peru has a highly successful track record of international mentorship because of the work of Drs. King Holmes from the University of Washington and Robert H. Gilman at the Johns Hopkins Bloomberg School of Public Health. Their combined efforts across 30+ years of residency and research in Peru have mentored and trained more than 20 Peruvian scientists at the PhD level and dozens of master’s graduates in Peru alone. More importantly, many of their more senior trainees have grown to become mentors themselves and are currently coaching new generations of Peruvian researchers. These scientists have led training grants and academic programs, mentored junior scientists, taken important public health positions, and some have even led important mentorship and research integrity capacity-building efforts. These scientists have also taken the wisdom and mentorship approach of their mentors from the global north, adapted it to LMIC settings, scaled their research operations and resources, and developed their own mentorship styles. Low- and middle-income country mentees do not merely clone and repeat what their mentors did: they transmit concepts and implement processes fitted to their own personalities and adapted through the lenses of local culture and beliefs. Thus, mentoring is customized and tailored to the needs, challenges, and barriers of LMICs. This is a replicable best practice, particularly in countries where senior and successful mentors from high-income country (HIC) universities have trained several generations of LMIC scientists who can evolve to become mentors themselves, creating sustainable, locally adapted mentoring capacities in LMICs.

### Gaps identified.

Four cases of plagiarism (*N* = 2) and cheating (*N* = 2) in a master’s program at UPCH led to the realization of the extent of research and academic misconduct events observed and the need for research integrity awareness and training.^9^ Greater attention was given to potential research misconduct events, and responsible conduct of research (RCR) training was enhanced, with curricula that included lectures on the role of mentoring as a mechanism to prevent misconduct. In addition, a mentoring program was established in 2012 at the U.S. Naval Medical Research Unit No. 6, in Lima to support the development of its Peruvian scientists and address an identified gap in the development of research capacity.

### Implementation strategies.

In 2013, UPCH developed an online research integrity course (CRI, for its acronym in Spanish), offered broadly at no charge to any interested participant.^10^ This course, funded with support by a grant supplement from the Fogarty International Center, includes a mentorship module that illustrates the importance and role of mentoring. Eleven short videos featuring senior UPCH investigators describe the qualities that mentors should have, the responsibilities of mentors, and their personal experiences with their respective mentees. Content was developed in part to address the previously described plagiarism and cheating cases observed in a local academic program and includes brief examinations and a completion diploma.^9^ In 2015, the Peruvian National Science and Technology Council (CONCYTEC for its acronym in Spanish) launched a new policy requiring CRI completion to apply for grant opportunities and be registered as investigators.^11,12^ In 2017, UPCH added the CRI completion requirement for students, faculty, and investigators applying to grants or requesting institutional review board (IRB) review.^10^ This free and effective online training course, in conjunction with a highly receptive leader at a key organization such as CONCYTEC, made CRI a central element of the RCR and mentoring training in the region.

### The mentorship environment today and next steps.

The “Mentoring the Mentor” workshop conducted in 2013 was an important element to catalyze interest and introduce mentoring as a concept to a large group of mid-career investigators and researchers at UPCH and other local South American institutions. Thousands of people have taken the CRI course, and the concept of mentoring is now well recognized and valued. Small-scale mentoring activities take place within research groups. Peer mentoring and progressive mentoring are slowly being introduced because of the lack of sufficient senior mentors. Several academic programs and scientific societies in South America now use CRI as a regular training and accreditation tool and as a result are better aware of their mentoring needs. All of these activities constitute the foundation of a future institutional mentoring program. Greater awareness of the value of mentoring programs among institutional authorities is needed, and explicit recognition of time invested in mentoring within academic responsibilities.

### Best practices.

Responsible conduct of research and mentoring training has become well known and sustainable in Peru because of the endorsement of these efforts from CONCYTEC. The involvement and uptake of the creation of the RCR training by the most important research sponsor in the country, particularly during a period of expanded research support, is a best practice that can be replicated by research-sponsoring institutions in LMICs. The development of a free-of-charge, online training module on RCR, including mentoring, is another best practice because it paves the way for scale-up and mandatory usage in multiple organizations and settings. The development of similar online modules with greater emphasis on training mentors, preparing potential mentees for mentorship relationships, and implementing mentoring programs in LMICs are areas for expansion. In parallel, the formal adoption and support of mentoring programs at LMIC institutions, as well as the formalization of institutional support for mentors and mentoring time, is needed. Academic programs and research groups that are already familiar and compliant with RCR training can be fertile grounds for implementation. A previous stage of adaptation of concepts to their local culture and beliefs is probably needed. Similarly, efforts should be initially tailored to the specific needs in each context, including the training of potential mentors, the scale of research activities, and available career growth opportunities for mentees, among other factors.

## Case 2—KEMRI

The Kenya Medical Research Institute is one of the leading biomedical research institutions in Africa. Constituting 320 researchers and 1,250 staff, KEMRI has made great contributions for the improvement of human health and quality of life through research, capacity building, innovation, and service delivery. The Kenya Medical Research Institute has major research collaborations with the Wellcome Trust, U.S. Centers of Disease Control, and numerous universities around the world.

### Mentorship infrastructure before the 2013 workshop.

Although mentorship programs have existed within some of these international collaborations, KEMRI on a whole has not institutionalized the practice. Formal mentorship has been a long unrealized goal of young investigators and scholars enrolled in KEMRI graduate education and research training. Formal assessments of early career professionals have indicated a need for mentors; these individuals cite the absence of mentorship as a barrier to their academic advancement. Initiatives to strengthen mentorship are met with the familiar challenge faced by many LMIC institutions of having few mentors in comparison to potential mentees. There has been no structured format for mentorship and annual performance work plans, and appraisals do not include elements of mentorship as a criterion for academic advancement.

### Gaps identified.

Following the “Mentoring the Mentors” workshop held in Mombasa, Kenya, for East African institutions, Dr. Cecilia Mbae was awarded a 1-year Clayton–Dedonder Mentorship Fellowship in 2015. She conducted a needs assessment on mentoring at the Centre for Microbiology Research (CMR). The needs assessment demonstrated a universal interest from scientists in mentoring, but only 40% reported prior experience as a mentee, and even fewer, 20%, had experience as a mentor. Furthermore, the following four gaps were identified as primary barriers to the institutionalization of a mentorship program at KEMRI: 1) lack of a well-established culture of mentorship; 2) lack of an approved formal policy on mentorship at CMR; 3) lack of an institution-wide policy on mentorship; and 4) lack of structured tools to evaluate mentorship.

### Implementation strategies.

Following the evaluation, two draft documents, a mentorship manual (inclusive of mentorship tools) and a draft mentorship policy, were developed. Two years later, these documents remain under institutional review for possible adoption. The manual details the process of mentor identification, how to develop and evaluate a mentorship plan, implementation of mentoring relationships, and termination of the mentorship relationship. It also provides documentation of best practices across KEMRI. In addition, the draft policy document suggests how to institutionalize mentorship at every level of KEMRI, establishes mentorship as a key deliverable in annual performance evaluations, and requires participation in mentorship for scientific advancement and promotion of research scientists.

### The mentorship environment today and next steps.

Based in part on the needs assessment, KEMRI scientists are collaborating with faculty from the University of California Global Health Institute and the University of Washington to advance these documents and formally establish an institution-wide mentoring program starting in mid-2018. The program aims to create a comprehensive, vigorous, and innovative mentorship training program to coach both mentors and mentees in skills relevant to effective mentoring and being an effective mentee. Trainings are planned for mentors and mentees modeled from the locally adapted “Mentoring the Mentor” workshops. In addition, this initiative plans to develop an Academy of Mentors at KEMRI to train and longitudinally track and highlight mentoring across KEMRI. Academy members will attend two annual in-person curriculum-based 2-day training workshops. In-person training will be reinforced via quarterly web-based “Mentor Consultation Clinics” to review ongoing mentee progress and challenges. Mentor outcomes (e.g., number of mentees, number of extramurally supported awards, promotion of mentors and mentees, mentor/mentee satisfaction) will be tracked over time.

### Best practice.

Performing a needs assessment early in the development phase of a mentoring program helps to galvanize support for mentoring among both faculty and institutional leadership; these are key ingredients to promote mentoring across an institution.

## Case 3—SJRI, India

Established in 2004, SJRI is one of the first independent research institutes in the country to be based in a private medical institution with independent administrative and research infrastructure. Saint John’s Research Institute, as an entity of St. John’s National Academy of Health Sciences (SJNAHS), joins the Medical College, a multi-specialty teaching hospital, the Nursing College, and the Institute of Allied Health and Hospital Administration, to advance health research and train future generations of scientists. The St. John’s Medical College graduates about 100 students each year. Saint John’s Research Institute provides a platform to complete the required dissertation for a portion of these students interested in developing academic careers in research. In addition, about 20 doctoral students per year pursue their PhD under the purview of SJRI.

### Mentorship infrastructure before the 2014 workshop.

Traditionally, all research “mentoring” within SJNAHS has been in the form of supervision by designated research “guides.” Before the “Mentoring the Mentors” workshop, there was no formal mentorship program for SJNAHS students. “Supervision by Guides” is mainly driven by standards established by the body governing medical education in India, the Medical Council of India and the local university, Rajiv Gandhi University of Health Sciences which confers the medical and doctoral degrees for SJNAHS.^13^ These institutional guidelines, although supporting educational objectives, do not include additional mentoring competencies and skills such as communication, goal setting, addressing diversity, and others, which distinguish mentoring from the more traditional supervisor–student relationship.^14^ Typically the guidelines specified by most universities in India only mention supervisors or guides as a requirement for postgraduates and doctoral students. The eligibility criteria for a PhD supervisor or guide is usually 3 years of experience in either teaching or research with at least three publications in peer reviewed journals.^15^ The teaching and research experience can vary between universities in India. Over the years, this has created a mind-set amongst faculty that holding a supervisor or guide role suffices for postgraduate and doctoral students. This is a significant barrier to overcome and careful strategies will be required to change this philosophy and practice.

### Gaps identified.

The India and South Asia “Mentoring the Mentors” Workshop identified various barriers to the institutionalization of mentorship within SJNAHS. These included: 1) the institutional approach—a lack of a general knowledge about the value of mentoring across the institution, 2) a mind-set that is fixated on a mere supervisory role driven by university and regulatory guidelines, 3) poor time management by mentors and mentees, 4) a lack of adequately trained research mentors, and 5) the absence of a structured mentorship guidance and policy framework.

### Implementation strategies.

Various mentorship integration strategies have been initiated at SJNAHS and SJRI. One, SJNAHS created the position of Vice-Dean to oversee postgraduate training and education to support improvements inclusive of mentorship. This position meets with a postgraduate student forum to inform guidelines which promote mentoring practices. Two, leaders across SJNAHS and SJRI have engaged informally with supervisors to sensitize them about the nuances between mentorship and supervision. Three, SJRI conducted a 2-day workshop for mentees to address research methodologies and mentor–mentee issues. Four, as a follow-up to the original workshop in 2014, Dr. Monica Gandhi from UCSF returned to SJRI in 2017 to hold another workshop to advance mentoring practices. Fifteen mentors from various departments in SJNAHS participated, some of whom were new to formal mentorship programs at the institution. Five, research methodology courses are now conducted at frequent intervals each year at SJRI where faculty, mentors, postgraduate students, and doctoral students are encouraged to participate. These courses provide an opportunity for mentees to interact with mentors and receive guidance on the design and implementation of their research and dissertation work. Last, SJRI initiated a PhD committee to both facilitate doctoral studies and create a foundation to the support mentorship program across the institution.

### The mentorship environment today and next steps.

The original workshop set the expectations and improved awareness about the value of creating a structured mentorship program across the institution. Seven mid- and senior-faculty from SJRI participated in the “Mentoring the Mentor” workshop, with four currently serving as mentors for eight doctoral students. These mentors cite the need for further improvement of mentor–mentee relationships and better time management. Overall, the workshop provided exposure to a diverse selection of topics that have enabled these mentors to increase their research productivity and mentorship activities. In addition, following the workshop, more SJNAHS faculty have applied for the India Alliance fellowships. These opportunities fund a specific research topic to support doctoral and postdoctoral fellows in research and thus provide an opportunity for mentoring young investigators. To date, five faculty from the medical college and SJRI have secured these prestigious awards. The ongoing collaborative relationship between University of California, San Francisco, and SJRI has provided the opportunity for young faculty and postdoctoral fellows to apply for the University of California Global Health Institute’s Fogarty Global Health Fellowships (GloCal). Since 2013, three young faculty have had the unique opportunity to be selected and mentored to conduct global health research in India. These GloCal Health Fellowships have provided unique exposure for mentees to be mentored by at least two experienced mentors from both University of California and St. John’s.

The year-long GloCal Health Fellowships have provided a unique research opportunity for early-career faculty to conduct research in both an interdisciplinary area and by an interdisciplinary team of mentors. This program has served as a foundation for best practices for successful mentor–mentee relationships for the future leaders and practitioners of global health. It has also helped fellows attain their short-term career goals and sensitize them to the benefits of a structured mentorship program.^16^

### Challenges to institutionalize mentorship programs.

One of the greatest challenges to institutionalize mentorship programs within SJRI is the current guidelines which only specify supervisor and guide roles; no formal documentation exists acknowledging the formal role of a mentor. This has created a mind-set among faculty that a supervision or guideship role will suffice. Inadequate awareness about the value of mentoring is another barrier. The current perception among supervisors is that mentorship requires investment of a lot more time and effort, and therefore many faculty are happy to keep a solely supervisory role. Last, at SJRI, the mentor and mentee do not get protected time for mentorship and research activities, competing demands result in poor time management and an incomplete mentoring experience.

Plans to expand mentoring within the Academy include the following: 1) Increase awareness of the value and benefits of mentoring among faculty through informal and formal engagements such as mentorship workshops and symposia for faculty and mentors; 2) encourage the PhD committee to promote, sensitize, and facilitate a structured mentorship program within SJNAHS and SJRI; 3) explore opportunities to garner institutional support to facilitate protected time for mentors and mentees; 4) encourage more researchers and faculty to apply for fellowships such as GloCal, India Alliance, and similar opportunities that will help foster mentorship; 5) apply for grant funding to support mentorship programs within the academy; and 6) investigate the opportunity to formally include the mentor role within university regulatory bodies.

### Best practices.

The “Mentoring the Mentors” workshop and subsequent symposium demonstrated the need for a continued push for awareness around the importance of mentorship. More of these programs will be required to overcome the present culture within the institution that fails to provide support for mentorship. Second, the GloCal and India Alliance fellowships have demonstrated that early-career faculty benefit immensely from a structured mentorship program. This highlights the need to create mentorship guidelines within the academy and to identify a pool of suitable mentors who are passionate about mentoring. Collaborations with universities in HIC, where mentorship programs are better recognized, have helped SJRI and SJNAHS to adopt best practices for mentorship and begin the sensitization process for local faculty on the importance and value of mentorship.

## Case 4—Universidade Eduardo Mondlane (UEM), Mozambique

The UEM, former Lourenço Marques University, was established in 1962 amidst Portuguese colonial rule. Until 2008, it was the only public medical school in the country and continues to stand as the leading academic institution in the country. Universidade Eduardo Mondlane hosts 143 faculty and graduates 70 undergraduates and 65 master’s and PhD students in the health sciences each year.

### Mentorship infrastructure before the 2016 workshop: before and after the medical education partnership (MEPI) initiative.

Since 2008, UEM and the University of California, San Diego (UCSD), have collaborated to develop a mentorship structure at UEM. In 2010, the MEPI, funded by the Office of Global AIDS Coordinator, National Institutes of Health, and Health Resources and Services Administration, was formed to develop, expand, and advance innovative models of medical education, including the advancement of mentoring capacity.^17,18^ The partnership between UEM and UCSD built a core group of dedicated faculty that also included participants from the Instituto de Higiene e Medicina Tropical from Universidade Nova de Lisboa (IHMT-UNL), Portugal, and the Universidade Federal da Bahia, Brazil, to specifically address the goals of mentorship development within MEPI in Mozambique. Additional support for mentorship capacity building was provided to other institutions in the country, including Universidade Lurio (UniLurio) and the Universidade Zambeze (UniZambeze). Before the UEM–UCSD partnership, the few faculty and researchers engaged in structured mentorship of early-career professionals were undertaken primarily as an informal one-on-one relationship between the mentee and the mentor without institutional support. With the expansion of UEM’s master’s and PhD programs, a significant need for mentoring at the institution exists. The collaboration provided a new foundation to train and mentor physicians and other health professionals through the provision of a structured peer mentorship program.

### Gaps identified.

A needs assessment and comprehensive analysis of the research environment in Mozambique’s major public universities was conducted to direct the next steps. This work identified barriers to and facilitators for the development of a research enterprise inclusive and supportive of mentorship. To facilitate access to mentorship, UEM formed a consortium with the Health Sciences faculty in two recently opened local universities, UniLurio and UniZambeze. The inclusion of these institutions was core to the development of the consortium as both are located in underserved regions of the country.

Despite successful mentoring relationships before the Southern Africa “Mentoring the Mentors” workshop in 2016, technical advice from partners was identified as an area of need. Additional meetings between the UCSD and Instituto de Higiene e Medicina Tropical- Universidade Nova de Lisboa (IHMT-UNL) faculties followed the workshop to address this barrier. In addition, participants from UEM at the workshop were sensitized to cultural, economic, and professional experience diversity among mentees that can impact the mentor–mentee relationship.

### The results—the mentorship environment today and next steps.

The evolution of these partnerships has supported the work of more than 63 new research projects, 19 of which received external funding and to date has resulted in the publication of more than 40 manuscripts.^19,20^ Several of these publications have Mozambican first authors representing a rise of first authorship from 29% in 2001–2010 to 38% from 2011 to 2013. The inclusion of UniLurio and UniZambeze was core to the development of the consortium as both are located in underserved regions of the country. Before inclusion in the mentorship program, the limited English fluency of the faculty and an absence of classes for advanced degrees were barriers for these institutions. As a result, master’s courses for this region of the country were selected based on local and national needs and government priorities. These were the first programs in the country to consider such factors before the implementation of new education programs. Furthermore, UniLurio graduated its inaugural master’s class of 45 students as a result of its inclusion in the mentorship core group.

In addition to the accomplishments in research and publications, knowledge generated through these endeavors has translated into health practice and policy. For example, two projects to develop point-of-care diagnostic devices, namely, point-of-care ultrasonography for diagnostic human immunodeficiencey virus (HIV) management and point-of-care CD4 cell enumeration for management of HIV coinfections, were implemented.^21^

Although UEM has made gains toward actualizing its strategic plan for improved research development infrastructure, it still lacks a well-formed program of mentorship that supports both the training of faculty and mentees. Universities included in the MEPI partnership are still challenged with 1) a shortage of skilled mentors; 2) an absence of infrastructure support for health professionals to mentor; 3) written and spoken English language fluency; 4) low salaries in the fields of research, academia, and health care, thus requiring multiple means of employment; and 5) significant delays in the institutional review board approval system, requiring 6 months or more to initiate a project.^20^ Complete commitment to this process can only be achieved with the support and understanding of senior faculty, administrative leaders, and other stakeholders on the critical role of the mentorship process and its influence on junior faculty career development and, in turn, on the importance of developing the next generation of faculty supported by a solid mentorship framework.

### Universidade Eduardo Mondlane best practices.

Following the 2016 “Mentoring the Mentor” workshop, the UEM mentorship program was established and a monthly group meeting of interested researchers ranging from undergraduate and PhD students with faculty was initiated. The group structure maximizes the influence and benefit of the relatively limited mentor pool while still providing opportunities for mentorship by senior faculty and peers. These monthly group mentoring sessions have resulted in 1) an increased interest in research, with a greater number of junior researchers and faculty members seeking support for mentorship; 2) a reinforced support system for mentees that facilitates collaborative work among mentees, thus increasing confidence to complete the work and a greater diversity of knowledge among mentees; and 3) a shift toward greater teamwork among the mentees, thus enabling mentees to act as mentors for the next generation of students.

## Discussion

These case studies reveal that regional mentoring workshops that target senior- and mid-career faculty from LMICs can catalyze the growth of mentorship initiatives across a diverse set of institutions across different regions of the world. As demonstrated in this article, mentorship programs vary greatly between countries in part because of resource availability and length of collaboration with local and international partners. In addition, advocacy that targets institutional leaders and senior faculty can play a central role in encouraging the establishment and enhancement of mentorship programs within these organizations.

The four presented case studies reveal the following best practices for LMIC institutions: 1) the integration of RCR practices is a critical component of mentor guidance and has the potential to expand to improving general mentoring practices through an online platform; 2) needs assessments can provide insights into the gaps and help strengthen support for mentorship at the institutional level; 3) support from leadership and formal recognition of the mentor’s role are crucial for true institutional integration of mentoring; 4) early-career faculty benefit immensely from fellowships that include structured mentorship; 5) group- and peer-mentorship models can facilitate mentoring, especially at institutions with few qualified mentors; 6) HIC institutions in collaboration with locally led initiatives can serve as catalysts and supporters of formal mentorship; 7) distance learning and other electronic platforms can be used to provide mentorship for research project development and implementation, teaching and clinical discussion, and didactic and research skills courses^22–24^; and 8) workshops, such as the “Mentoring the Mentors” series, can catalyze a transition for individuals and institutions to uncover both their personal and institutional influences as mentors.

Overall, mentoring programs across many LMIC institutions continue to progress albeit at different paces. Regardless of the stage of a mentoring program within an institution, the implementation of best practices, such as the ones presented in this article, can help to further advance mentoring initiatives that, in time, will further develop the careers of mentees, leading to improved health around the globe. Furthermore, these cases can serve as examples that highlight best practices that can be adapted and emulated in other settings in LMICs. Last, the role of institutions is critical to change the culture of mentoring through developing supportive policies, serving as examples, providing required resources, and encouraging mentoring as an essential element of academic success and achievement.
